# Habitual sleep duration and spontaneous intracerebral hemorrhage: a prospective UK Biobank cohort study with supporting hospital-based neurosurgical data

**DOI:** 10.3389/fneur.2026.1960098

**Published:** 2026-10-26

**Authors:** Weichuan Dai, Yuyan Zhu, Liming Yang, Mingming Zhang, Yingxian Chen, Zhongning Fang, Zhen Qi, Pengwei Hou, Xieli Guo

**Affiliations:** 1 Department of Neurosurgery, Jinjiang Municipal Hospital (Shanghai Sixth People’s Hospital Fujian), Jinjiang, Fujian, China; 2 Department of Cardiovascular Surgery, The First Hospital of Jilin University, Changchun, Jilin, China; 3 Department of Neurosurgery, The Second Xiangya Hospital, Central South University, Changsha, Hunan, China

**Keywords:** inflammatory, long sleep, prospective cohort study, sICH, sleep duration

## Abstract

**Background:**

Abnormal sleep duration has been associated with stroke, but evidence specific to spontaneous intracerebral hemorrhage (sICH) remains limited.

**Methods:**

We conducted a two-part observational study. The prospective UK Biobank analysis included 314,236 participants free of sICH at baseline, among whom 1,387 developed incident sICH. The supporting cross-sectional hospital analysis included 312 patients with imaging-confirmed sICH and 515 non-ICH neurosurgical controls from Jinjiang Municipal Hospital. Habitual sleep duration was categorized as short (<7 h/day), normal (7–9 h/day), or long (>9 h/day). Cox proportional hazards models [hazard ratios (HRs)] and logistic regression [odds ratios (ORs)] were used, together with restricted cubic splines, additional adjustment for medications, blood pressure, and indicators of prevalent ill health, and multiple imputation of missing data. A systemic inflammatory marker score and interaction analyses with false-discovery-rate correction were examined as secondary, exploratory analyses.

**Results:**

Compared with normal sleep, short and long sleep were associated with higher incident sICH risk in the UK Biobank [adjusted HRs 1.14 (95% CI 1.03–1.26) and 1.55 (1.15–2.09)]; the short-sleep association was modest and less robust in sensitivity analyses. Corresponding cross-sectional ORs in the hospital dataset were 1.49 (1.04–2.13) and 2.05 (1.17–3.59), respectively. Restricted cubic splines suggested U-shaped associations in both datasets, with the lowest estimated hazard or odds at approximately 7–8 h/day. Estimates were similar after additional confounder adjustment (HRs 1.10 and 1.43 for short and long sleep, respectively) and multiple imputation (HRs 1.13 and 1.50, respectively). No interaction, including with inflammatory marker burden, remained significant after false-discovery-rate correction, and adjustment for the marker score changed the estimates only modestly.

**Conclusion:**

Both short and long sleep duration were associated with higher incident sICH risk in the UK Biobank and higher odds of sICH in the hospital dataset, with stronger and more consistent associations observed for long sleep. Systemic inflammatory markers neither explained nor reliably modified these associations, and because long sleep may partly reflect prevalent ill health, the long-sleep association warrants cautious interpretation. Sleep duration may be a useful marker of sICH susceptibility.

## 1 Introduction

Spontaneous intracerebral hemorrhage (sICH) is one of the most severe forms of stroke and is associated with high risks of early death, long-term disability, and recurrent vascular events. Current global estimates indicate that intracerebral hemorrhage accounts for nearly one-third of incident strokes and almost half of stroke-related deaths and disability-adjusted life-years worldwide (1). Although hypertension and other established vascular risk factors remain central to sICH prevention, the effectiveness of available preventive strategies is limited, and a substantial proportion of events cannot be fully explained by conventional risk factors (2). Identifying additional modifiable behavioral factors and the biological pathways through which they may influence sICH risk is therefore of considerable public health and clinical importance.

Sleep duration has emerged as an important component of cardiovascular and cerebrovascular health. A recent meta-analysis of 43 cohort studies reported a U-shaped association between sleep duration and stroke, with both short and long sleep associated with higher stroke incidence and mortality (3). Meta-analyses of prospective cohorts have generally reported larger and more consistent excess risks for long than for short sleep, with pooled relative risks of 1.65 versus 1.15 (4) and 1.45 versus 1.15 (5) for fatal or nonfatal stroke. Nevertheless, most previous studies have focused on total or ischemic stroke, and evidence specific to intracerebral hemorrhage remains comparatively limited. In a prospective study of 409,156 Chinese adults, more than 43,000 incident strokes, including 8,128 intracerebral hemorrhages, were documented during approximately 9 years of follow-up; both very short and very long sleep were associated with higher risks of intracerebral hemorrhage compared with intermediate sleep duration (6). In another prospective study of 79,881 Swedish adults, 826 intracerebral hemorrhages occurred during a mean follow-up of 14.6 years. Short sleep was associated with intracerebral hemorrhage, whereas long sleep was more clearly associated with total and ischemic stroke; accompanying genetic analyses did not provide consistent evidence for a causal effect of sleep duration on stroke (7). These differences may reflect variation in study populations, sleep-duration definitions, outcome ascertainment, event numbers, and control of confounding, highlighting the need for large studies specifically focused on sICH.

Low-grade systemic inflammation is one biologically plausible link between abnormal sleep duration and cerebrovascular vulnerability. Chronic systemic inflammation contributes to endothelial dysfunction, metabolic dysregulation, vascular injury, and several major age-related diseases (8). A systematic review and meta-analysis of 72 studies involving more than 50,000 adults found that sleep disturbance and prolonged sleep duration were associated with higher circulating inflammatory markers, particularly C-reactive protein and interleukin-6 (9). Similarly, a cross-sectional analysis of 459,796 UK Biobank participants demonstrated that blood biomarker profiles, including C-reactive protein, varied according to habitual sleep duration (10). In mice, sleep fragmentation increases the bone-marrow production of inflammatory monocytes and accelerates atherosclerosis (11), and endothelial dysfunction, blood–brain barrier leakage, and inflammation are recognized features of the cerebral small vessel disease that underlies most sICH (12, 13). In the context of intracerebral hemorrhage, activation of peripheral immune cells and neuroinflammatory pathways contributes to blood–brain barrier disruption, perihematomal edema, and secondary brain injury (14). However, inflammatory biomarkers in most clinical ICH studies are measured after hemorrhage onset and may predominantly reflect the acute response to brain injury; for example, plasma C-reactive protein rises markedly over the first 48 to 72 h after sICH (15). Consequently, it remains uncertain whether a pre-existing systemic inflammatory marker burden lies on the pathway between habitual sleep duration and sICH, modifies this association, or is merely a correlated marker of poor vascular health.

In the present study, we combined a large population-based prospective cohort from the UK Biobank with supporting data from a hospital-based neurosurgical sample from Jinjiang Municipal Hospital. We examined the categorical and nonlinear associations of habitual sleep duration with incident sICH in the prospective cohort and evaluated whether a convergent cross-sectional pattern was present in a clinically ascertained hospital population. As secondary and exploratory aims, we examined whether the associations differed across major clinical characteristics, including systemic inflammatory marker burden, and how the estimates changed after additional adjustment for a composite score derived from C-reactive protein, white blood cell count, neutrophil-to-lymphocyte ratio, and systemic immune-inflammation index. Because the available data could not establish the temporal ordering of sleep, inflammatory markers, and sICH, we considered three non-exclusive possibilities: that inflammatory marker burden mediates part of the association, that it modifies the association, or that it is a correlated risk marker without a causal role. By integrating longitudinal population-based evidence with complementary clinical data, we aimed primarily to clarify the association between abnormal sleep duration and sICH and, secondarily, to explore whether systemic inflammatory markers explained or modified this relationship.

## 2 Methods

### 2.1 Study design and data sources

We conducted a two-part observational study using data from the UK Biobank and a single-center hospital-based clinical dataset from Jinjiang Municipal Hospital (Shanghai Sixth People’s Hospital Fujian). The UK Biobank component was designed as a prospective cohort analysis evaluating the association of habitual sleep duration with incident sICH. The hospital component was designed as a retrospective cross-sectional clinical analysis to examine whether comparable sleep–sICH patterns were present in a neurosurgical population; it was regarded as supporting clinical evidence rather than as an independent population-based replication.

The UK Biobank is a large population-based prospective cohort that recruited more than 500,000 adults from the United Kingdom between 2006 and 2010 and collected questionnaire data, physical measurements, biological samples, and linked health-record information for long-term follow-up (16). The present study was designed and reported in accordance with recommendations for observational epidemiologic studies and studies using routinely collected health data (17, 18). UK Biobank (UKB) data were accessed under application number 240510. UKB has approval from the North West Multi-centre Research Ethics Committee as a Research Tissue Bank (REC reference 11/NW/0382, renewed in 2016 as 16/NW/0274 and in 2021 as 21/NW/0157), and all participants provided written informed consent.

The hospital-based study used de-identified routinely collected clinical data from adult neurosurgical admissions between January 2022 and December 2025. Clinical information was abstracted from admission records, nursing histories, laboratory databases, neuroimaging reports, discharge diagnoses, and inpatient problem lists. The hospital-based study was approved by the Ethics Committee of Jinjiang Municipal Hospital (approval No. jjsyyll-2026-189), which waived the requirement for individual informed consent because only de-identified data collected during routine care were analyzed retrospectively; the study was conducted in accordance with the Declaration of Helsinki. The hospital component was intended to complement the prospective cohort analysis by evaluating the same sleep duration, sICH outcome, and inflammatory markers in a clinically ascertained neurosurgical population. The participant-selection process for both datasets is shown in Figure 1.

**Figure 1:**
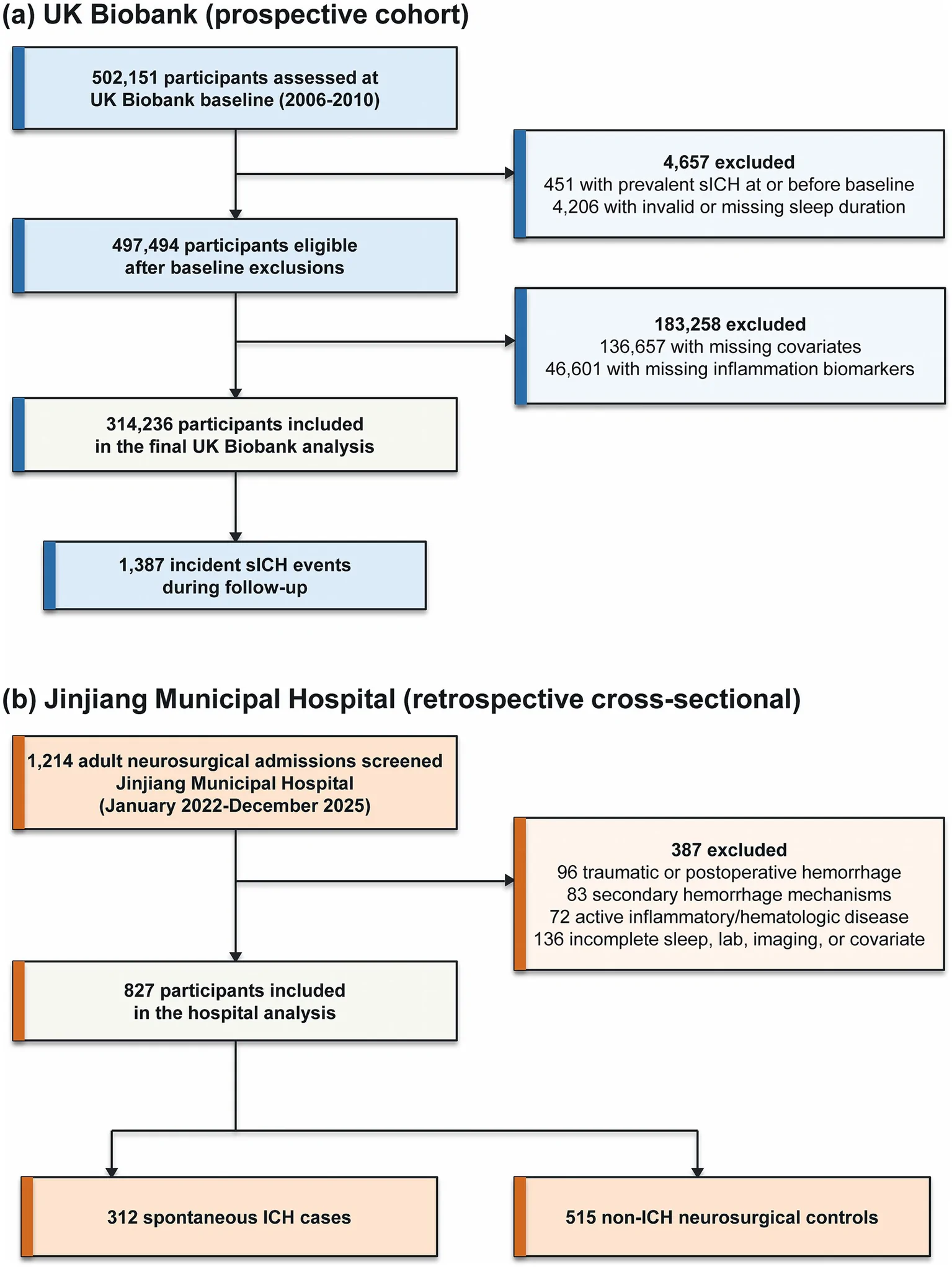
Flowchart of participant selection in the UK Biobank and Jinjiang Municipal Hospital study. Panel **(a)** shows selection of the UK Biobank cohort. Of 502,151 participants assessed at baseline, 314,236 were included after exclusions, with 1,387 incident sICH events during follow-up. Panel **(b)** shows selection of the hospital-based sample. Of 1,214 neurosurgical admissions screened, 827 were included, comprising 312 spontaneous ICH cases and 515 non-ICH controls. sICH, spontaneous intracerebral hemorrhage; ICH, intracerebral hemorrhage.

### 2.2 Study population

In the UK Biobank, 502,151 participants were initially screened. We excluded participants with prevalent sICH at or before baseline (*n* = 451), invalid or missing sleep duration (*n* = 4,206), missing core covariates required for multivariable adjustment (*n* = 136,657), and missing inflammation biomarkers required to construct the systemic inflammatory marker score (*n* = 46,601). The final UKB analytic sample included 314,236 participants who were free of sICH at baseline, among whom 1,387 developed incident sICH during follow-up.

For the hospital-based dataset, 1,214 adult neurosurgical admissions were screened. We excluded patients with traumatic or postoperative hemorrhage (*n* = 96); aneurysmal subarachnoid hemorrhage, vascular malformation, tumor-related hemorrhage, hemorrhagic transformation, or other secondary hemorrhage mechanisms (*n* = 83); active infection, systemic inflammatory disease, hematologic disease, or recent major surgery that could substantially influence inflammatory biomarkers (*n* = 72); and incomplete sleep, laboratory, imaging, or covariate data (*n* = 136). The final hospital sample included 827 patients, comprising 312 patients with spontaneous ICH and 515 non-ICH neurosurgical controls.

Non-ICH controls were adult neurosurgical patients admitted during the same period without imaging or diagnostic evidence of spontaneous intracerebral hemorrhage at the index admission; their principal diagnoses comprised brain tumors without hemorrhage (*n* = 141, 27.4%), traumatic brain injury without intracranial hemorrhage (*n* = 63, 12.2%), degenerative spinal disease (*n* = 119, 23.1%), functional neurosurgical disorders (trigeminal neuralgia or hemifacial spasm; *n* = 84, 16.3%), hydrocephalus (*n* = 31, 6.0%), unruptured cerebrovascular disorders (*n* = 36, 7.0%), and other conditions (*n* = 41, 8.0%). Because the hospital component was retrospective and cross-sectional, it was used to assess clinical consistency of the exposure–outcome pattern rather than to estimate incident risk. Moreover, because both cases and controls were drawn from neurosurgical admissions, the hospital estimates may be affected by admission-related (Berkson-type) selection bias (19).

### 2.3 Sleep duration

In the UK Biobank, sleep duration was obtained from the baseline touchscreen questionnaire. Participants reported their usual sleep duration in hours per 24-h period. In the hospital dataset, habitual pre-admission sleep duration was abstracted from medical records and nursing admission histories. For patients with sICH, the sleep-duration record was intended to reflect habitual sleep before the index hemorrhage rather than sleep during acute hospitalization. Other sleep characteristics, including sleep quality, insomnia, daytime napping, shift work, sleep regularity, and obstructive sleep apnea, were not incorporated because they were not comparably available in the two datasets.

Sleep duration was categorized as short sleep (<7 h/day), normal sleep (7–9 h/day), and long sleep (>9 h/day). Normal sleep duration was used as the reference group. This categorization was selected because 7–9 h is broadly consistent with adult sleep-duration recommendations and because previous epidemiologic studies have evaluated short and long sleep in relation to stroke and stroke subtypes (6, 7). In addition to categorical analyses, sleep duration was modeled as a continuous variable in dose–response analyses. Because short and long sleep may be related to inflammation and sICH through different mechanisms, they were analyzed as separate contrasts throughout rather than combined into a single deviation-from-normal score, with the pre-specified normal range of 7–9 h/day as the reference (7, 20).

### 2.4 Outcome ascertainment

The primary outcome was spontaneous intracerebral hemorrhage. In the UK Biobank, incident sICH was identified from linked health records using the UKB intracerebral hemorrhage date information. Participants were followed from baseline assessment until the first recorded sICH event, death, loss to follow-up, or December 31, 2022, whichever occurred first. Participants with sICH recorded at or before baseline were excluded as prevalent cases.

Linked health-record data have been widely used for stroke ascertainment in large-scale cohort studies, including UK Biobank, although careful phenotype definition is particularly important for stroke subtypes (21, 22). In the hospital dataset, sICH was defined as spontaneous nontraumatic intraparenchymal hemorrhage confirmed by computed tomography or magnetic resonance imaging. This definition was aligned with contemporary clinical concepts of spontaneous ICH and excluded hemorrhage due to trauma, postoperative bleeding, aneurysm, vascular malformation, tumor, hemorrhagic transformation, or other secondary causes (2).

### 2.5 Systemic inflammatory marker burden

Systemic inflammatory marker burden was assessed using circulating inflammatory biomarkers. In the UK Biobank, biomarkers were measured at baseline. In the hospital dataset, laboratory values were taken from the first available admission blood sample obtained before major neurosurgical intervention whenever such a sample was available. Because hospital biomarkers were measured during the index admission, after sICH onset in cases, they could reflect both pre-existing inflammatory burden and an acute response to hemorrhage (15) and cannot be regarded as antecedent to sICH; therefore, all hospital-based inflammation-related analyses were considered exploratory and descriptive.

White blood cell count, neutrophil count, lymphocyte count, platelet count, and C-reactive protein were used to characterize systemic inflammatory status. These biomarkers were selected because chronic low-grade inflammation is increasingly recognized as a pathway linking behavioral, metabolic, vascular, and age-related disease processes (8). Sleep duration has also been associated with inflammatory biomarkers, supporting the biological plausibility of evaluating inflammation in the sleep–vascular disease relationship (9, 23).

We derived three cell-count-based inflammatory indices: neutrophil-to-lymphocyte ratio, platelet-to-lymphocyte ratio, and systemic immune-inflammation index. The neutrophil-to-lymphocyte ratio was calculated as neutrophil count divided by lymphocyte count, the platelet-to-lymphocyte ratio as platelet count divided by lymphocyte count, and the systemic immune-inflammation index as platelet count multiplied by neutrophil count and divided by lymphocyte count. These indices are accessible markers of systemic inflammatory and immune status and have been evaluated in stroke and intracerebral hemorrhage research (24, 25).

A systemic inflammatory marker score was calculated separately within each dataset as the mean of standardized log-transformed C-reactive protein, standardized white blood cell count, standardized log-transformed neutrophil-to-lymphocyte ratio, and standardized log-transformed systemic immune-inflammation index. Log transformation was applied to right-skewed biomarkers before standardization, and each component was standardized using the dataset-specific mean and SD. The score was intended to capture a shared inflammatory signal across complementary components, namely the hepatic acute-phase response (C-reactive protein), the total leukocyte count, and the balance of innate immune cells, lymphocytes, and platelets (neutrophil-to-lymphocyte ratio and systemic immune-inflammation index), while reducing the random variability of any single marker, in line with established composite scores of low-grade inflammation (26). Equal weights were chosen *a priori* for simplicity and transparency; because the neutrophil-to-lymphocyte ratio and the systemic immune-inflammation index share neutrophil and lymphocyte counts, this weighting gives the neutrophil–lymphocyte axis greater implicit weight, and each marker was therefore also examined separately in sensitivity analyses. Higher values indicated greater systemic inflammatory marker burden. High marker burden was defined as the top quartile of this score within each dataset; participants below the top quartile were classified as having low/intermediate marker burden. The continuous score was used for additional adjustment in Model IV, whereas the high-burden category was used for descriptive and subgroup analyses.

### 2.6 Covariates

Covariates were selected *a priori* based on clinical relevance, previous epidemiologic evidence, and conceptual considerations linking sleep duration, inflammation, vascular risk, and spontaneous ICH. Covariates included age, sex, race/ethnicity in the UK Biobank, body mass index, educational attainment, income level, smoking status, alcohol drinking status, physical activity, Townsend deprivation index, urban/rural residence, Charlson comorbidity index, diabetes, and hypertension (27).

Age was modeled as a continuous variable. Sex was categorized as female or male. Body mass index was modeled as a continuous variable. Educational attainment was categorized as college or above, upper secondary, below upper secondary, or unknown. Income level was categorized as low, intermediate, or high. Smoking status was categorized as never, former, current, or unknown. Alcohol drinking status was categorized as never, former, current, or unknown. Physical activity was categorized as adequate or inadequate. Residence was categorized as urban or rural.

Townsend deprivation index was available only in the UK Biobank and was dichotomized as ≤0 versus >0, with higher values indicating greater material deprivation. Race/ethnicity was included in the UK Biobank models where applicable. Clinical covariates included Charlson comorbidity index, diabetes, and hypertension. The Charlson comorbidity index was calculated only from diagnoses recorded before the start of follow-up. In the UK Biobank, it was derived from illnesses self-reported at the baseline verbal interview and hospital inpatient diagnoses dated before the baseline assessment, using all inpatient records available before baseline (from 1997 in England, 1981 in Scotland, and 1998 in Wales), with ICD-9 and ICD-10 codes mapped according to the algorithms of Quan and colleagues (28); diagnoses recorded after baseline, including the incident sICH event itself, did not contribute. In the hospital dataset, the index was derived from comorbidities documented before the index admission, excluding the index cerebrovascular event and its sequelae, such as hemiplegia. Hypertension, diabetes, and comorbidity burden were included because vascular and cardiometabolic risk factors are central to ICH susceptibility and may also be related to sleep duration and systemic inflammation (2).

For the hospital dataset, covariates were harmonized where possible using corresponding clinical record variables, including demographic characteristics, residence, body mass index, smoking and alcohol history, comorbidities, and admission-record information. Variables not comparably available in the hospital dataset, such as Townsend deprivation index, were not included in hospital models.

### 2.7 Statistical analysis

Baseline characteristics were summarized according to variable type and distribution. Continuous variables were first evaluated for approximate normality. Variables with approximately normal distributions were presented as mean (SD), whereas skewed continuous variables were presented as median (interquartile range). Categorical variables were summarized as number (%). In the UK Biobank, characteristics were described according to incident sICH status during follow-up. In the hospital dataset, characteristics were described for non-ICH neurosurgical controls and patients with spontaneous ICH. Formal hypothesis testing for baseline characteristics was not emphasized because these comparisons were intended for descriptive characterization rather than covariate selection.

In the UK Biobank, Cox proportional hazards models were used to estimate hazard ratios (HRs) and 95% confidence intervals (CIs) for incident sICH. Follow-up time was calculated from baseline assessment to incident sICH, death, loss to follow-up, or administrative censoring. In the hospital dataset, logistic regression models were used to estimate odds ratios (ORs) and 95% CIs for sICH status. Because the hospital component was cross-sectional, ORs were interpreted as associations with imaging-confirmed sICH rather than incident risk estimates; accordingly, the term risk is used only for the prospective UK Biobank estimates.

Four sequential models were fitted. Model I adjusted for age and sex. Model II additionally adjusted for sociodemographic factors, including educational attainment, income level, residence, and dataset-specific socioeconomic variables; race/ethnicity and Townsend deprivation category were included in the UK Biobank where available. Model III further adjusted for lifestyle and clinical covariates, including body mass index, smoking status, alcohol drinking status, physical activity, Charlson comorbidity index, diabetes, and hypertension. Model IV additionally included the continuous systemic inflammatory marker score. Model III was used as the principal adjusted model because the marker score could act as a confounder, a mediator, or a correlated marker of poor health in the UK Biobank and, in the hospital dataset, could partly be a consequence of sICH; adjustment for such a variable may introduce overadjustment bias (29). Model IV was therefore interpreted only as a descriptive additional adjustment. Analyses were performed separately for short sleep and long sleep, each compared with normal sleep duration.

To assess potential nonlinearity, sleep duration was modeled as a continuous variable using restricted cubic splines with four knots placed at the 5th, 35th, 65th, and 95th percentiles of the dataset-specific sleep-duration distribution. Separate spline models were fitted in the UK Biobank and hospital dataset, using Cox regression for the UKB analysis and logistic regression for the hospital analysis. The reference value was set at 7 h/day. The displayed sleep-duration range was restricted to 4–11 h/day to avoid unstable inference at sparsely populated extremes. Nonlinearity was assessed using a Wald test for the nonlinear spline terms. Restricted cubic splines were used because they provide a flexible approach for modeling nonlinear exposure–outcome relationships in epidemiologic data (30).

Subgroup and interaction analyses were conducted in a two-step manner. First, we systematically assessed potential effect modification by testing interactions between sleep duration category and each pre-specified candidate modifier. Candidate modifiers comprised all covariates included in Model III and systemic inflammatory marker burden, with dataset-specific variables tested where available (15 candidate modifiers in the UK Biobank and 13 in the hospital dataset). Multiplicative interaction was assessed by adding cross-product terms between sleep duration category and one candidate modifier at a time to the Model III pre-inflammation model. For each candidate modifier, the overall interaction was evaluated using a joint Wald test of all cross-product terms between the three-category sleep variable and the modifier. Second, stratified estimates were presented for variables showing nominal evidence of interaction (P for interaction <0.05); because this selection was data-driven, the stratified estimates were regarded as descriptive. Based on these interaction tests, stratified analyses were conducted for inflammatory marker burden, hypertension, and age group in the UK Biobank, and for age group in the hospital-based dataset; stratified estimates by inflammatory marker burden were not presented for the hospital dataset because the markers were measured after sICH onset. High marker burden was defined as the top quartile of the systemic inflammatory marker score within each dataset, and the remaining participants were classified as having low/intermediate marker burden. Age was categorized as <60 years and ≥60 years. Within each subgroup, short sleep and long sleep were separately compared with normal sleep duration. Stratified models retained the Model III adjustment set except that the stratifying variable was omitted. To account for multiple testing, interaction *p* values were additionally adjusted across all candidate modifiers within each dataset using the Benjamini–Hochberg false-discovery-rate procedure (31). All subgroup findings were regarded as hypothesis-generating, particularly for hospital-based strata with limited sample size and wide confidence intervals. All nominal and Benjamini–Hochberg-adjusted interaction *p* values are provided in Supplementary Table S1.

After fitting the sequential regression models, we described the change in the log-effect estimates after additional adjustment for the systemic inflammatory marker score. Specifically, the percentage change in the log-effect estimate was calculated by comparing Model III, which included demographic, sociodemographic, lifestyle, and clinical covariates but not the marker score, with Model IV, which additionally included the continuous systemic inflammatory marker score:

$$\frac{\ln(\text{Model}\;\mathrm{III}\;\text{estimate})-\ln(\text{Model}\;\mathrm{IV}\;\text{estimate})}{\ln(\text{Model}\;\mathrm{III}\;\text{estimate})}\times 100\%$$

For the UK Biobank, this calculation was based on HRs; for the hospital-based dataset, it was based on ORs. A positive value indicated that the sleep–sICH association was smaller after additional adjustment for the marker score. This change-in-estimate quantity is purely descriptive: it cannot distinguish whether the marker score acts as a confounder, a mediator, a correlated marker, or, in the hospital dataset, a consequence of sICH, and it therefore does not represent the proportion of the association explained by inflammation (32).

Formal counterfactual mediation analysis was not performed. Natural direct and indirect effects require the mediator to be measured after the exposure and before the outcome and assume no unmeasured exposure–outcome, exposure–mediator, or mediator–outcome confounding (33, 34); these conditions could not be met because sleep duration and inflammatory markers were measured at the same baseline assessment in the UK Biobank and inflammatory markers were measured after sICH onset in the hospital dataset.

Several pre-specified sensitivity analyses and model diagnostic assessments were conducted to evaluate the robustness and validity of the main findings. To reduce potential reverse causation, the fully adjusted UK Biobank analyses (Model IV) were repeated in a 3-year landmark framework by excluding participants who developed sICH within the first 3 years after baseline and recalculating follow-up from the 3-year landmark until incident sICH, death, loss to follow-up, or December 31, 2022, whichever occurred first, while retaining the same exposure definitions, covariates, and Cox model specifications. To examine whether the findings depended on the construction of the composite systemic inflammatory marker score, Model IV was refitted by replacing the composite score with individual inflammatory markers, including C-reactive protein, neutrophil-to-lymphocyte ratio, platelet-to-lymphocyte ratio, and systemic immune-inflammation index, with right-skewed markers log-transformed and standardized before analysis; analyses were also repeated using high marker burden, defined as the top quartile of the dataset-specific composite score, as a binary covariate. The robustness of outcome definition was assessed in the UK Biobank using a broader incident hemorrhagic stroke endpoint based on linked hospital and mortality records, including ICD-10 codes I60 to I62, after excluding participants with the corresponding diagnosis at or before baseline; these three sensitivity analyses were based on Model IV. The proportional hazards assumption for the UK Biobank Cox models was evaluated using covariate-specific and global tests based on scaled Schoenfeld residuals. For the most extensively adjusted hospital-based logistic regression model (Model IV), goodness of fit was assessed using the Hosmer–Lemeshow test based on deciles of predicted probability; calibration was evaluated using calibration plots and the calibration intercept and slope, which equal 0 and 1 by construction in the development sample, so that optimism-corrected values were estimated by internal validation with 1,000 bootstrap resamples; discrimination was summarized by the area under the receiver operating characteristic curve with DeLong 95% CIs, and overall accuracy by the Brier score; multicollinearity was assessed using generalized variance inflation factors (GVIF^(1/(2 × Df))^), with values greater than 2.24 (equivalent to a variance inflation factor of 5) considered potentially important; and influential observations were identified using leverage values, standardized Pearson and deviance residuals, and Cook’s distance (threshold 4/n), with models refitted after excluding observations flagged by pre-specified thresholds and re-estimated with HC3 robust standard errors to evaluate the stability of the estimates. Finally, to assess the influence of control selection, the principal hospital model (Model III) was refitted after restricting controls to elective admissions for degenerative spinal disease, trigeminal neuralgia, or hemifacial spasm, thereby excluding diagnoses that could independently affect sleep, systemic inflammatory markers, or acute stress. To address potential confounding by intracerebral hemorrhage-specific risk factors and prevalent ill health, Model III was additionally adjusted in the UK Biobank for baseline use of antiplatelet agents and oral anticoagulants (35), antihypertensive medication, and statins and for measured systolic blood pressure (extended model 1), and then additionally for self-rated health, depression, and frailty, the last defined using a modified Fried phenotype (36) (extended model 2); because blood pressure and its treatment may lie on the pathway between sleep duration and sICH, these models were regarded as sensitivity analyses rather than as the principal analysis. To evaluate the impact of missing data, eligible participants excluded because of missing covariates or biomarkers were compared with included participants with respect to age, sex, sleep-duration distribution, and sICH incidence, and Models III and IV were re-estimated in all eligible participants after multiple imputation by chained equations with 20 imputed datasets, including the event indicator and the Nelson–Aalen estimate of the cumulative hazard in the imputation model (37); estimates were combined using Rubin’s rules.

All analyses were performed using R software (version 4.3.2; R Foundation for Statistical Computing, Vienna, Austria). Statistical tests were two-sided, and *p* values <0.05 were considered statistically significant.

## 3 Results

### 3.1 Characteristics of study participants

Table 1 summarizes the characteristics of participants according to sICH status in the UK Biobank (at baseline) and the hospital-based dataset (at the index admission). Among 314,236 UK Biobank participants free of sICH at baseline, 1,387 developed incident sICH during follow-up, representing 0.4% of the analytic cohort, whereas 312,849 remained free of sICH. The hospital-based dataset comprised 827 neurosurgical patients, including 312 patients with imaging-confirmed spontaneous ICH and 515 non-ICH neurosurgical controls.

**Table 1:** Characteristics of participants in the UK Biobank (at baseline) and the hospital-based dataset (at the index admission).

Characteristic	UK Biobank (*n* = 314,236)		Hospital-based dataset (*n* = 827)	
	non-sICH (*n* = 312,849)	incident sICH (*n* = 1,387)	controls (*n* = 515)	sICH (*n* = 312)
Age, years	56.6 (8.0)	61.6 (6.5)	56.8 (12.4)	62.9 (11.3)
Sex, *n* (%)				
Female	170,603 (54.5)	633 (45.6)	278 (54.0)	137 (43.9)
Male	142,246 (45.5)	754 (54.4)	237 (46.0)	175 (56.1)
BMI, kg/m^2^	27.6 (4.8)	27.6 (4.9)	24.6 (3.6)	25.1 (3.8)
Sleep duration group, *n* (%)				
Short sleep, <7 h/day	77,417 (24.7)	366 (26.4)	130 (25.2)	96 (30.8)
Normal sleep, 7–9 h/day	229,833 (73.5)	979 (70.6)	364 (70.7)	191 (61.2)
Long sleep, >9 h/day	5,599 (1.8)	42 (3.0)	21 (4.1)	25 (8.0)
Smoking status, *n* (%)				
Never	167,232 (53.5)	647 (46.6)	276 (53.6)	127 (40.7)
Former	110,295 (35.3)	559 (40.3)	112 (21.7)	82 (26.3)
Current	34,466 (11.0)	177 (12.8)	127 (24.7)	103 (33.0)
Unknown	856 (0.3)	4 (0.3)	0 (0.0)	0 (0.0)
Alcohol drinking status, *n* (%)				
Never	12,587 (4.0)	68 (4.9)	246 (47.8)	174 (55.8)
Former	11,163 (3.6)	72 (5.2)	53 (10.3)	41 (13.1)
Current	288,876 (92.3)	1,245 (89.8)	216 (41.9)	97 (31.1)
Unknown	223 (0.1)	2 (0.1)	0 (0.0)	0 (0.0)
Educational attainment, *n* (%)				
College or above	139,144 (44.5)	602 (43.4)	214 (41.6)	103 (33.0)
Upper secondary	104,029 (33.3)	418 (30.1)	181 (35.1)	115 (36.9)
Below upper secondary	68,282 (21.8)	360 (26.0)	120 (23.3)	94 (30.1)
Unknown	1,394 (0.4)	7 (0.5)	0 (0.0)	0 (0.0)
Income level, *n* (%)				
Low	158,749 (50.7)	880 (63.4)	240 (46.6)	182 (58.3)
Intermediate	139,699 (44.7)	471 (34.0)	225 (43.7)	111 (35.6)
High	14,401 (4.6)	36 (2.6)	50 (9.7)	19 (6.1)
Physical activity, *n* (%)				
Adequate	292,067 (93.4)	1,279 (92.2)	411 (79.8)	208 (66.7)
Inadequate	20,782 (6.6)	108 (7.8)	104 (20.2)	104 (33.3)
Townsend deprivation index, *n* (%)				
≤0	222,979 (71.3)	1,000 (72.1)	NA	NA
>0	89,870 (28.7)	387 (27.9)	NA	NA
Residence, *n* (%)				
Urban	270,129 (86.3)	1,210 (87.2)	362 (70.3)	234 (75.0)
Rural	42,720 (13.7)	177 (12.8)	153 (29.7)	78 (25.0)
Charlson comorbidity index	0.00 (0.00–1.00)	1.00 (0.00–2.00)	0.00 (0.00–1.00)	1.00 (0.00–2.00)
Diabetes, *n* (%)				
No	294,487 (94.1)	1,235 (89.0)	453 (88.0)	244 (78.2)
Yes	18,362 (5.9)	152 (11.0)	62 (12.0)	68 (21.8)
Hypertension, *n* (%)				
No	225,579 (72.1)	814 (58.7)	361 (70.1)	116 (37.2)
Yes	87,270 (27.9)	573 (41.3)	154 (29.9)	196 (62.8)
White blood cell count, ×10^9^/L	6.9 (2.2)	7.0 (2.4)	6.7 (1.9)	7.4 (2.2)
Platelet count, ×10^9^/L	253.8 (60.2)	245.8 (60.4)	240.5 (58.4)	236.2 (61.7)
C-reactive protein, mg/L	1.36 (0.67–2.82)	1.46 (0.74–3.10)	1.58 (0.72–3.20)	2.45 (1.10–5.36)
Neutrophil-to-lymphocyte ratio	2.15 (1.67–2.78)	2.31 (1.74–3.04)	2.05 (1.54–2.78)	2.70 (1.88–3.84)
Systemic immune-inflammation index, ×10^9^/L	532.87 (396.00–720.59)	546.57 (400.77–747.24)	495.0 (355.0–692.0)	640.0 (455.0–915.0)
Inflammatory marker score	0.0 (0.7)	0.1 (0.7)	−0.1 (0.6)	0.2 (0.7)
High inflammatory marker burden, top quartile, *n* (%)				
No	234,709 (75.0)	968 (69.8)	401 (77.9)	211 (67.6)
Yes	78,140 (25.0)	419 (30.2)	114 (22.1)	101 (32.4)

Values are presented as mean (SD) for approximately normally distributed continuous variables, median (interquartile range) for skewed continuous variables, or number (%), as appropriate. The UK Biobank analysis included participants free of spontaneous intracerebral hemorrhage at baseline and was stratified according to incident sICH status during follow-up. The hospital-based dataset comprised non-ICH neurosurgical controls and patients with imaging-confirmed spontaneous ICH from Jinjiang Municipal Hospital (Shanghai Sixth People’s Hospital Fujian). Sleep duration was categorized as short sleep (<7 h/day), normal sleep (7–9 h/day), and long sleep (>9 h/day). The inflammatory marker score was calculated within each dataset as the mean of standardized log-transformed C-reactive protein, standardized white blood cell count, standardized log-transformed neutrophil-to-lymphocyte ratio, and standardized log-transformed systemic immune-inflammation index. High inflammatory marker burden was defined as the top quartile of this score within each dataset; in the hospital dataset, inflammatory markers were measured at admission, after sICH onset in cases. Townsend deprivation index was available only in the UK Biobank. sICH denotes spontaneous intracerebral hemorrhage; ICH, intracerebral hemorrhage; BMI, body mass index. The Charlson comorbidity index was calculated only from diagnoses recorded before baseline in the UK Biobank and before the index admission in the hospital dataset, excluding the index event.

In both datasets, participants with sICH were older and more frequently male than those without sICH. In the UK Biobank, the mean age was 61.6 years among participants who developed incident sICH, compared with 56.6 years among those who did not, and the proportion of men was 54.4% versus 45.5%, respectively. A similar pattern was observed in the hospital dataset, in which patients with sICH had a mean age of 62.9 years compared with 56.8 years among controls, and 56.1% of sICH patients were male compared with 46.0% of controls. Mean BMI was similar between incident sICH and non-sICH participants in the UK Biobank, whereas hospital-based sICH patients had a slightly higher mean BMI than controls.

Sleep-duration distributions differed by sICH status in both study populations. In the UK Biobank, participants who developed incident sICH were more likely to report short sleep (26.4% vs. 24.7%) or long sleep (3.0% vs. 1.8%) and were less likely to report normal sleep duration (70.6% vs. 73.5%) than those who remained free of sICH. These differences were more pronounced in the hospital dataset, where short sleep was reported by 30.8% of sICH patients compared with 25.2% of controls, and long sleep by 8.0% compared with 4.1%, whereas normal sleep was less common among sICH patients (61.2% vs. 70.7%).

Participants with sICH also generally had less favorable socioeconomic, behavioral, and clinical profiles. In both datasets, the sICH groups had higher proportions of current smoking, lower educational attainment, low income, and inadequate physical activity. Comorbidity burden was greater among participants with sICH, as reflected by higher Charlson comorbidity index values and higher prevalences of diabetes and hypertension. The difference in hypertension was particularly marked in the hospital dataset, where 62.8% of sICH patients had hypertension compared with 29.9% of controls; corresponding proportions in the UK Biobank were 41.3 and 27.9%.

Inflammatory marker profiles were also less favorable among participants with sICH. White blood cell count, C-reactive protein, neutrophil-to-lymphocyte ratio, systemic immune-inflammation index, and the composite inflammatory marker score were generally higher in the sICH groups in both datasets, whereas platelet counts were slightly lower. High marker burden, defined as the top quartile of the dataset-specific marker score, was present in 30.2% of participants with incident sICH compared with 25.0% of non-sICH participants in the UK Biobank, and in 32.4% of hospital-based sICH patients compared with 22.1% of controls; in the hospital dataset, these markers were measured after sICH onset and may partly reflect the acute response to hemorrhage. Overall, participants with sICH were characterized by older age, a greater burden of vascular and systemic comorbidities, a higher prevalence of non-optimal sleep duration, and higher inflammatory marker levels.

### 3.2 Association between sleep duration and sICH in the UK Biobank and Jinjiang Municipal Hospital study

Figure 2 summarizes the associations of sleep duration with sICH across the four sequentially adjusted models, using normal sleep duration of 7–9 h/day as the reference. In the UK Biobank, both short and long sleep were associated with a higher risk of incident sICH. In Model I, adjusted for age and sex, the HRs were 1.22 (95% CI, 1.10–1.35) for short sleep and 1.78 (95% CI, 1.32–2.39) for long sleep. The estimates were progressively attenuated after additional adjustment for sociodemographic, lifestyle, and clinical characteristics. In Model III, the principal adjusted model, short and long sleep remained associated with incident sICH, with HRs of 1.14 (95% CI, 1.03–1.26) and 1.55 (95% CI, 1.15–2.09), respectively. After further adjustment for the continuous systemic inflammatory marker score in Model IV, the corresponding estimates were 1.10 (95% CI, 1.01–1.20) for short sleep and 1.42 (95% CI, 1.06–1.90) for long sleep.

**Figure 2:**
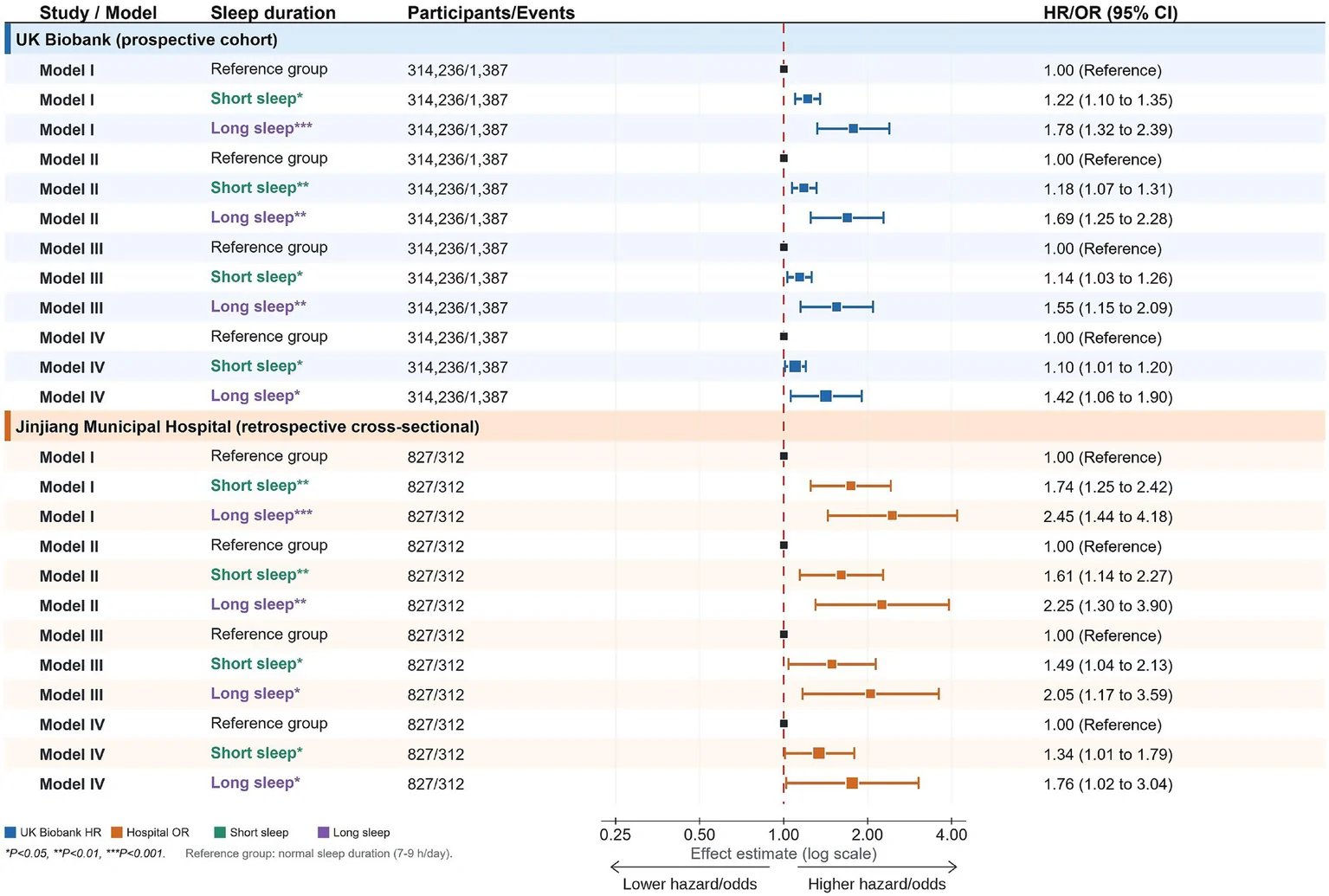
Associations of sleep duration with sICH across sequentially adjusted models in the UK Biobank and Jinjiang Municipal Hospital study. Estimates are presented as hazard ratios (HRs) with 95% CIs for incident sICH in the UK Biobank and odds ratios (ORs) with 95% CIs for sICH in the hospital-based analysis. Normal sleep duration (7–9 h/day) was the reference group. Model I adjusted for age and sex; Model II additionally adjusted for sociodemographic factors; Model III further adjusted for lifestyle and clinical covariates; and Model IV additionally adjusted for the continuous systemic inflammatory marker score. Model III was the principal adjusted model; in the hospital dataset, Model IV adjusted for inflammatory markers measured after sICH onset and is shown for completeness only. Squares indicate effect estimates and horizontal lines indicate 95% CIs; arrows indicate a lower or higher hazard (UK Biobank) or odds (hospital dataset). ^*^p < 0.05, ^**^p < 0.01, ^***^p < 0.001. sICH, spontaneous intracerebral hemorrhage.

A similar cross-sectional pattern was observed in the hospital-based analysis, although the magnitude of the associations was generally greater and the confidence intervals were wider. In Model I, short sleep was associated with higher odds of sICH (OR, 1.74; 95% CI, 1.25–2.42), while long sleep showed a stronger association (OR, 2.45; 95% CI, 1.44–4.18). After adjustment for the full set of demographic, socioeconomic, lifestyle, and clinical covariates in Model III, the ORs were 1.49 (95% CI, 1.04–2.13) for short sleep and 2.05 (95% CI, 1.17–3.59) for long sleep. Additional adjustment for the inflammatory marker score in Model IV, which in this dataset was measured after sICH onset, yielded ORs of 1.34 (95% CI, 1.01–1.79) and 1.76 (95% CI, 1.02–3.04), respectively. Across both datasets and all sequential models, long sleep consistently showed larger effect estimates than short sleep, whereas the short-sleep estimates were modest and their lower confidence limits approached unity after additional adjustment.

Figure 3 presents the restricted cubic spline analyses of sleep duration and sICH. Significant nonlinear associations were observed in both datasets, with a U-shaped exposure–response pattern in the UK Biobank (*P* for nonlinearity = 0.004) and the hospital-based study (*P* for nonlinearity = 0.018). The lowest estimated hazard (UK Biobank) or odds (hospital dataset) occurred at approximately 7–8 h/day, with progressively higher estimates at both shorter and longer sleep durations. The increase was particularly pronounced at longer sleep durations, especially in the hospital dataset. Confidence intervals widened toward the extremes of the sleep-duration distribution, reflecting lower precision among participants reporting very short or very long sleep. Overall, the spline findings were consistent with the categorical analyses and supported a nonlinear association between non-optimal sleep duration and sICH.

**Figure 3:**
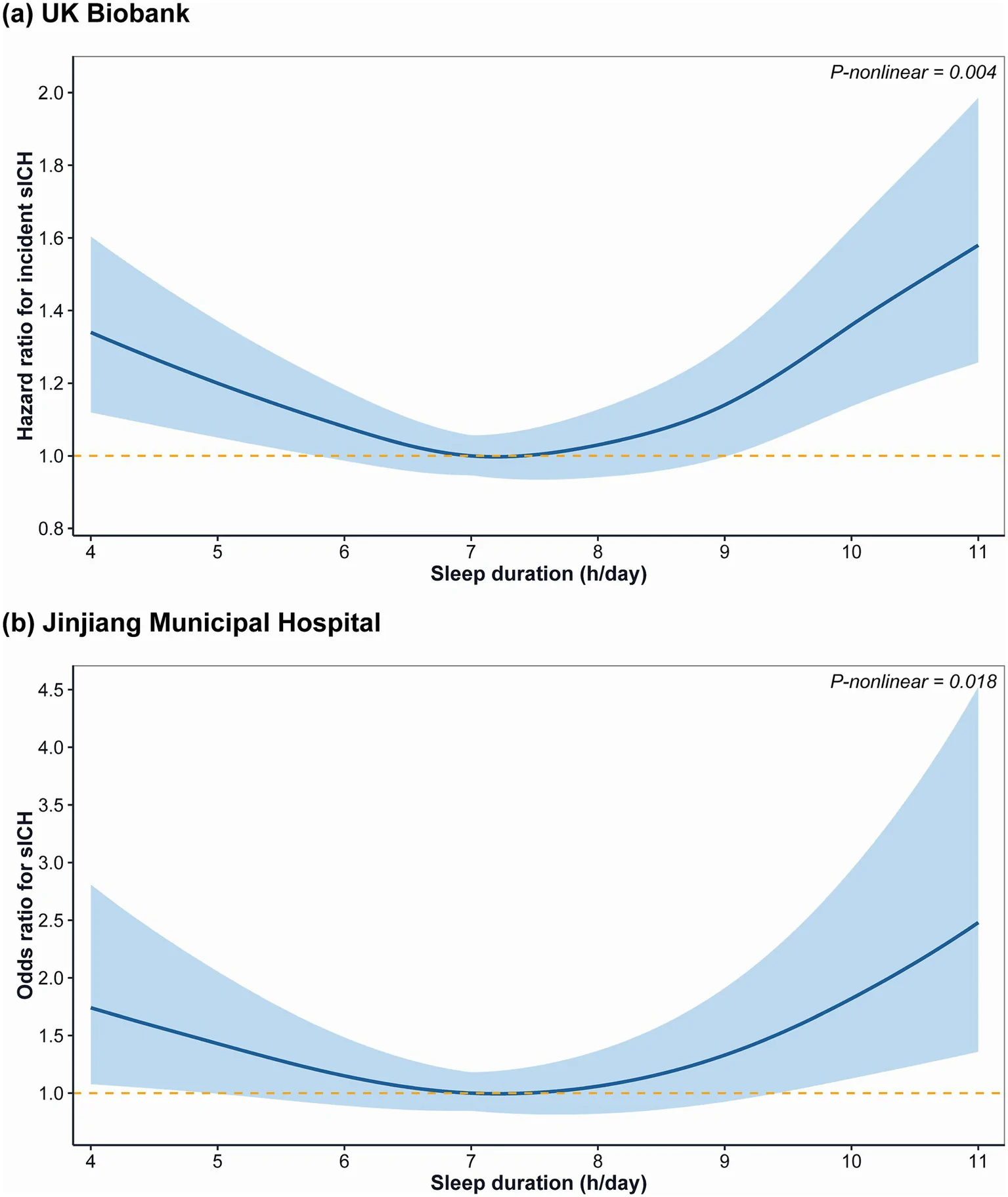
Restricted cubic spline associations between sleep duration and sICH in the UK Biobank and Jinjiang Municipal Hospital study. Panel **(a)** shows adjusted hazard ratios for incident sICH in the UK Biobank, and Panel **(b)** shows adjusted odds ratios for sICH in the hospital-based analysis. Solid lines indicate effect estimates, shaded areas indicate 95% CIs, and the dashed horizontal line denotes HR/OR = 1.00. Sleep duration was modeled from 4 to 11 h/day using 7 h/day as the reference. *p* values for nonlinearity were obtained using Wald tests. sICH, spontaneous intracerebral hemorrhage; HR, hazard ratio; OR, odds ratio; CI, confidence interval.

### 3.3 Subgroup analyses of the association between sleep duration and sICH

Figure 4 presents subgroup analyses using normal sleep duration (7–9 h/day) as the reference within each stratum. In the UK Biobank, nominally significant interactions were observed for inflammatory marker burden (*P* for interaction = 0.008), hypertension (*P* for interaction = 0.015), and age (*P* for interaction = 0.037); none remained significant after Benjamini–Hochberg correction across the candidate modifiers (all adjusted *p* ≥ 0.113; Supplementary Table S1); because these three modifiers were selected for stratified presentation on the basis of nominal interaction, the stratified estimates below are descriptive. Among participants with low/intermediate marker burden, neither short sleep (HR, 1.05; 95% CI, 0.94–1.18) nor long sleep (HR, 1.26; 95% CI, 0.90–1.78) was clearly associated with incident sICH, whereas both associations were evident among those with high marker burden (short sleep: HR, 1.27; 95% CI, 1.08–1.49; long sleep: HR, 1.96; 95% CI, 1.19–3.24). Similarly, no clear associations were observed among participants without hypertension, while short and long sleep were associated with higher sICH risk among those with hypertension (HR, 1.31; 95% CI, 1.12–1.53 and HR, 1.86; 95% CI, 1.22–2.84, respectively). Associations were also concentrated among participants aged ≥60 years, with HRs of 1.23 (95% CI, 1.07–1.42) for short sleep and 1.73 (95% CI, 1.19–2.52) for long sleep, whereas neither association was evident among those aged <60 years.

**Figure 4:**
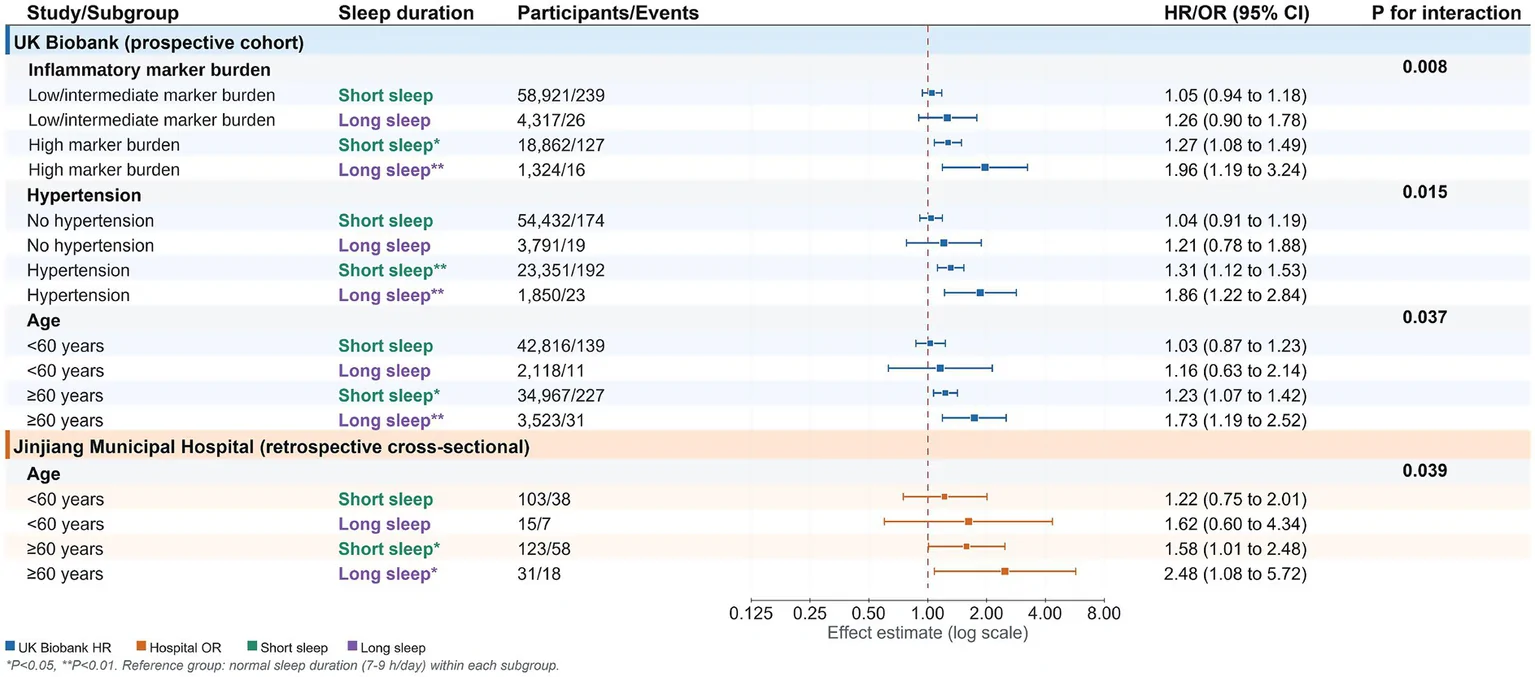
Subgroup associations of sleep duration with sICH in the UK Biobank and Jinjiang Municipal Hospital study. Subgroup analyses were conducted according to inflammatory marker burden, hypertension, and age in the UK Biobank, and according to age in the hospital-based dataset. Normal sleep duration (7–9 h/day) was the reference within each subgroup. Estimates are presented as HRs with 95% CIs for incident sICH in the UK Biobank and ORs with 95% CIs for sICH in the hospital analysis. Models were adjusted for the Model III covariates, excluding the stratifying variable. High marker burden was defined as the top quartile of the UK Biobank inflammatory marker score; hospital strata by inflammatory marker burden are not shown because the markers were measured after sICH onset. The modifiers shown were selected on the basis of nominal interaction (*p* < 0.05), so the stratified estimates are descriptive. *p* values for interaction are nominal; Benjamini–Hochberg-adjusted *p* values for all candidate modifiers are provided in Supplementary Table S1, and none remained significant after correction. All subgroup findings are hypothesis-generating. Squares indicate effect estimates and horizontal lines indicate 95% CIs. ^*^p < 0.05, ^**^p < 0.01. sICH, spontaneous intracerebral hemorrhage; HR, hazard ratio; OR, odds ratio; CI, confidence interval.

In the hospital-based analysis, nominal interactions were observed for admission inflammatory marker burden (*P* for interaction = 0.041) and age (*P* for interaction = 0.039), neither of which remained significant after false-discovery-rate correction (both adjusted *p* = 0.267; Supplementary Table S1); because hospital inflammatory markers were measured after sICH onset, stratified estimates by marker burden are not presented. No clear associations were observed among patients aged <60 years, while among those aged ≥60 years, short sleep was associated with higher odds of sICH (OR, 1.58; 95% CI, 1.01–2.48) and long sleep showed a stronger association (OR, 2.48; 95% CI, 1.08–5.72). Overall, long sleep generally showed larger effect estimates than short sleep across strata; however, none of the subgroup differences was robust to correction for multiple testing, and the wide confidence intervals in several hospital-based strata warrant particular caution.

### 3.4 Change in estimates after additional adjustment for the inflammatory marker score

Additional adjustment for the continuous systemic inflammatory marker score was accompanied by smaller estimates for both short and long sleep in the two datasets. In the UK Biobank, the HR for short sleep decreased from 1.14 in Model III to 1.10 in Model IV, corresponding to a 27.3% change on the log-effect scale (95% CI, 5.7 to 48.8%). The HR for long sleep decreased from 1.55 to 1.42, corresponding to a change of 20.0% (95% CI, −2.6 to 42.5%). In the hospital-based analysis, the OR for short sleep decreased from 1.49 to 1.34 after additional adjustment for the marker score, corresponding to a change of 26.6% (95% CI, 4.1 to 49.1%), whereas the OR for long sleep decreased from 2.05 to 1.76, corresponding to a change of 21.2% (95% CI, −4.8 to 47.3%); because these markers were measured after sICH onset, the hospital values are shown for completeness only. These percentages describe changes in regression coefficients and do not indicate the proportion of the associations explained by inflammation.

### 3.5 Sensitivity analyses and model diagnostics

The primary findings remained broadly consistent across the pre-specified sensitivity analyses. In the 3-year landmark analysis based on Model IV, exclusion of participants who developed sICH during the first 3 years of follow-up resulted in only modest attenuation relative to the corresponding full-cohort estimates (1.10 and 1.42), with HRs of 1.09 (95% CI 1.00 to 1.19) for short sleep and 1.39 (95% CI 1.02 to 1.89) for long sleep in the UK Biobank (Supplementary Table S2; Supplementary Text S2); the lower confidence limit for short sleep reached unity. Replacing the composite marker score with C-reactive protein, neutrophil-to-lymphocyte ratio, platelet-to-lymphocyte ratio, systemic immune-inflammation index, or high marker burden yielded directionally consistent estimates in both datasets, with long sleep consistently showing the larger association (Supplementary Tables S3A,B; Supplementary Text S3). When a broader hemorrhagic stroke endpoint defined by ICD-10 codes I60 to I62 was used, short and long sleep remained associated with higher risk, with HRs of 1.08 (95% CI 1.02 to 1.15) and 1.34 (95% CI 1.10 to 1.63), respectively (Supplementary Table S4; Supplementary Text S4). No evidence of violation of the proportional hazards assumption was observed for short sleep, long sleep, or the global UK Biobank Cox model (global *p* = 0.28; Supplementary Table S5; Supplementary Text S5). The most extensively adjusted hospital logistic model demonstrated acceptable goodness of fit (Hosmer–Lemeshow *χ^2^* = 7.18, *df* = 8; *p* = 0.517) and moderate discrimination (area under the receiver operating characteristic curve 0.781, 95% CI 0.745 to 0.816; Brier score 0.176); the apparent calibration intercept and slope were 0 and 1 by construction, and the optimism-corrected values after 1,000 bootstrap resamples were −0.036 and 0.918, respectively (Supplementary Table S6 and Supplementary Figures S1, S2; Supplementary Text S6). All generalized variance inflation factors were below 2.2, and exclusion of eight potentially influential observations, as well as re-estimation with HC3 robust standard errors, produced only minor changes in the estimates for short and long sleep, indicating that multicollinearity and individual influential observations were unlikely to explain the findings (Supplementary Tables S7, S8 and Supplementary Figure S3; Supplementary Text S7). When controls were restricted to 203 elective patients with degenerative spinal disease, trigeminal neuralgia, or hemifacial spasm (515 participants, including 312 sICH cases), the Model III ORs for short and long sleep were 1.47 (95% CI 0.96 to 2.26) and 2.18 (95% CI 1.13 to 4.20), respectively, directionally consistent with the principal estimates, although the confidence interval for short sleep included unity (Supplementary Tables S9, S10; Supplementary Text S8). In the UK Biobank, additional adjustment for antithrombotic and antihypertensive treatment, statins, and systolic blood pressure yielded HRs of 1.12 (95% CI 1.01 to 1.24) for short sleep and 1.48 (95% CI 1.10 to 2.00) for long sleep, and further adjustment for self-rated health, depression, and frailty yielded 1.10 (95% CI 0.99 to 1.23) and 1.43 (95% CI 1.04 to 1.98), respectively, so that the confidence interval for short sleep included unity in the most extensively adjusted model (Supplementary Table S11; Supplementary Text S9). Compared with the 314,236 included participants (mean age 56.6 years; short sleep 24.8%; long sleep 1.8%; sICH incidence 0.32 per 1,000 person-years), the 183,258 eligible participants excluded because of missing covariates or biomarkers had a mean age of 57.8 years, a short-sleep prevalence of 26.8%, a long-sleep prevalence of 3.0%, and an sICH incidence of 0.46 per 1,000 person-years (Supplementary Table S12; Supplementary Text S10). After multiple imputation in all 497,494 eligible participants, the Model III HRs were 1.13 (95% CI 1.05 to 1.22) for short sleep and 1.50 (95% CI 1.21 to 1.87) for long sleep, similar to the complete-case estimates (Supplementary Table S13; Supplementary Text S10). Overall, the sensitivity and diagnostic analyses supported the main results, although the short-sleep association was less robust than the long-sleep association.

## 4 Discussion

To our knowledge, this study provides the first integrated evidence combining a large prospective analysis of incident spontaneous intracerebral hemorrhage with supporting hospital-based clinical data, and its main finding beyond the well-established U-shaped pattern is that long sleep showed a larger and more consistent association than short sleep, which persisted after additional adjustment for intracerebral hemorrhage-specific confounders and indicators of prevalent ill health. In the UK Biobank, short and long sleep were associated with 14 and 55% higher hazards of incident sICH, respectively, after multivariable adjustment (10 and 42% after additional adjustment for the marker score). The hospital analysis showed a convergent cross-sectional pattern, with short and long sleep associated with 49 and 105% higher odds of imaging-confirmed sICH, respectively. Restricted cubic spline analyses suggested a U-shaped association in both populations, with the lowest estimated hazard or odds at approximately 7 to 8 h of sleep per day and a steeper increase at longer durations. Exploratory subgroup analyses did not provide robust evidence of effect modification, as none of the interactions survived correction for multiple testing. Additional adjustment for the marker score changed the UK Biobank estimates by approximately 20 to 27% on the log scale, a descriptive change in estimate rather than a proportion explained by inflammation. Collectively, these findings indicate that nonoptimal sleep duration, particularly long sleep, is associated with sICH across population-based and clinical settings, but they do not establish systemic inflammation as a causal intermediate or a reliable modifier.

Our findings are broadly consistent with previous evidence relating abnormal sleep duration to stroke, while extending that evidence to a more specific hemorrhagic phenotype. Mc Carthy and colleagues applied conditional logistic regression to 4,496 matched participants in the international INTERSTROKE study, including 1,799 patients with ischemic stroke and 439 with intracerebral hemorrhage. Compared with intermediate sleep duration, sleeping less than 5 h was associated with acute stroke at an OR of 3.15 (95% CI 2.09 to 4.76), while sleeping more than 9 h was associated with an OR of 2.67 (95% CI 1.89 to 3.78) (38). The larger estimates than those observed in our prospective analysis may reflect the use of more extreme sleep thresholds, retrospective ascertainment of recent sleep, inclusion of multiple stroke subtypes, and potential changes in sleep preceding an acute event. In a prospective study of 31,750 Chinese adults with a mean age of 61.7 years, Zhou and colleagues found that sleeping at least 9 h was associated with incident total stroke at an HR of 1.23 (95% CI 1.07 to 1.41), whereas sleeping less than 6 h was not clearly associated with stroke. Poor sleep quality was also associated with a 56% higher risk of hemorrhagic stroke, suggesting that prolonged sleep may partly capture fragmented or nonrestorative sleep (39). For intracerebral hemorrhage specifically, the China Kadoorie Biobank reported HRs of 1.19 for 5 h or less and 1.23 for 10 h or more relative to 7 to 8 h (6), whereas a Swedish cohort found an association of short sleep with intracerebral hemorrhage (HR 1.21) and of long sleep mainly with total and ischemic stroke (7). Our UK Biobank estimate for long sleep (HR 1.55) is closer to pooled estimates for total stroke (1.45 to 1.65) than to these ICH-specific estimates, whereas our short-sleep estimate (HR 1.14) is similar to the pooled estimate for short sleep (1.15) (4, 5); a multinational cohort likewise observed a J-shaped pattern for deaths and major cardiovascular events, with an HR of 1.41 for more than 10 h but a non-significant HR of 1.09 for 6 h or less (40). Differences across studies may therefore arise from sleep thresholds and reference categories, inclusion of daytime napping, sleep quality, population age and ethnicity, outcome definitions and subtype adjudication, the number of hemorrhagic events, and the degree to which long sleep reflects prevalent ill health. Beyond confirming the U-shaped pattern, the present study adds evidence by focusing specifically on incident sICH, by showing that the excess hazard was larger and more consistent for long than for short sleep, and by showing that adjustment for systemic inflammatory markers did not materially explain the associations.

The inflammation analyses were secondary and require cautious interpretation. In the sequential models, additional adjustment for the continuous marker score changed the log-hazard ratios by 27.3% for short sleep and 20.0% for long sleep in the UK Biobank. However, the confidence interval for long sleep included zero, and such changes in regression coefficients cannot distinguish confounding, mediation, and suppression (32); they should not be interpreted as the proportions of the associations explained by inflammation. We did not perform a formal mediation analysis because inflammatory markers were not measured between sleep assessment and sICH in either dataset, and combining short and long sleep in a single deviation score could produce an artifactual inverse indirect effect if the two extremes relate differently to inflammation. The present pattern is therefore more consistent with inflammatory markers acting as correlated indicators of vascular vulnerability than with inflammation lying on a causal pathway or reliably modifying the association, and it provides no evidence that inflammation protects against sICH. Independent evidence nonetheless supports inflammation as a marker of cerebrovascular vulnerability. In a nested case–control analysis that included 4,476 incident ICH cases and 5,285 controls, Karim and colleagues found that each SD higher usual log-transformed C-reactive protein was associated with ICH at an OR of 1.22 (95% CI 1.10 to 1.36) (41). Among 13,929 middle-aged and older Chinese adults followed for a median of 8.28 years, Xu and colleagues found that the highest versus lowest quartile of the systemic immune-inflammation index was associated with hemorrhagic stroke at an HR of 1.60 (95% CI 1.03 to 2.49) (42). Sleep itself may influence this inflammatory environment. In 614 adults assessed using questionnaires and polysomnography, Patel and colleagues reported that each additional hour of habitual sleep was associated with approximately 8% higher C-reactive protein and 7% higher interleukin-6, while shorter objectively measured sleep was associated with higher tumor necrosis factor-α (23). Experimentally, sleep fragmentation increases the production of inflammatory monocytes through a hypothalamic–bone marrow axis (11). Potential pathways include sympathetic and hypothalamic–pituitary–adrenal activation, loss of normal nocturnal blood pressure reduction, oxidative stress, endothelial dysfunction, leukocyte activation, altered platelet activity, and impaired cerebral small-vessel integrity (12). A systematic review of 24 adult human studies also concluded that short sleep, sleep restriction, and sleep outside the recommended range were associated with impaired macrovascular or microvascular endothelial function (43). At the same time, inflammation measured after hemorrhage may reflect the consequence rather than the cause of brain injury. Among 9,589 patients with acute ICH, Wang and colleagues found that C-reactive protein above 10 mg/L was associated with severe ICH at an OR of 1.64 (95% CI 1.46 to 1.84) and in-hospital death at an OR of 2.03 (95% CI 1.39 to 2.95) (44), and plasma C-reactive protein increases markedly over the first 48 to 72 h after sICH (15). This acute response is particularly relevant to the hospital component of our study, in which inflammatory markers were measured after sICH onset; the hospital inflammation-related estimates therefore cannot be interpreted as reflecting pre-existing inflammation.

The modifiers presented were selected because they showed nominal interaction, none of the interactions remained significant after correction for multiple testing, and the hospital inflammatory markers were measured after sICH onset. The descriptively stronger associations among UK Biobank participants with high inflammatory marker burden, hypertension, or age of at least 60 years, and among older hospital patients, are therefore hypothesis-generating rather than evidence of effect modification. The hypertension interaction is supported by a prospective study of 1,268 hypertensive adults in which Eguchi and colleagues found that sleeping less than 7.5 h was associated with incident stroke at an HR of 2.21 (95% CI 1.30 to 3.76) (45). Age-related findings have been less uniform. In 95,023 stroke-free Chinese adults followed for a mean of 7.9 years, Song and colleagues documented 3,135 strokes, including 631 hemorrhagic events. Sleeping more than 8 h was associated with total stroke at an HR of 1.29 (95% CI 1.01 to 1.64), with a stronger association among older participants at an HR of 1.47 (95% CI 1.05 to 2.07); among women, the corresponding HR for hemorrhagic stroke was 3.58 (95% CI 1.28 to 10.06) (46). Conversely, Joundi and colleagues followed 82,795 Canadian adults and recorded 1,705 strokes, finding that sleep of at least 10 h was associated with stroke among participants younger than 70 years at an HR of 2.29 (95% CI 1.04 to 5.06), but not among older participants (47). In another analysis of 6,877 Chinese adults with 136 incident strokes over approximately four years, Fang and colleagues found that sleep of 6 h or less was associated with stroke at an HR of 1.65 (95% CI 1.04 to 2.61), with stronger patterns among participants aged 45 to 65 years and those with elevated blood pressure (48). These differences indicate that age modification may depend on the sleep cutoff, stroke phenotype, comorbidity distribution, survival selection, and precision within individual strata. Therefore, our interaction results should be regarded as hypothesis-generating (49), particularly because multiple modifiers were examined, none of the interactions remained significant after false-discovery-rate correction, and several hospital strata contained few long sleepers or sICH cases.

From a clinical perspective, our findings suggest that simple screening of sleep habits may be reasonable in high-risk populations, such as older adults and individuals with hypertension. Sleep health is now recognized as a core component of cardiovascular health, and current consensus recommendations identify at least 7 h of regular nightly sleep as necessary for optimal adult health, with approximately 7 to 9 h generally regarded as an appropriate target (50, 51). Nevertheless, the present observational findings do not establish that deliberately shortening or extending sleep will prevent sICH. Instead, short or, in particular, long sleep should be regarded as an accessible clinical marker that may prompt evaluation of blood pressure control, sleep quality, obstructive sleep apnea, depression, frailty, medication use, and chronic cardiometabolic or inflammatory conditions. This may be especially relevant for patients with resistant or poorly controlled hypertension, among whom professional guidance supports assessment for obstructive sleep apnea because of its close relationships with sympathetic activation, hypertension, and cerebrovascular disease (52). Such screening should complement rather than replace established sICH prevention strategies, particularly sustained blood pressure detection, treatment, and control, which remain central to reducing the risk of first and recurrent intracerebral hemorrhage (2).

## 5 Strengths and limitations

This study has several strengths. The prospective UK Biobank component included more than 300,000 participants free of sICH at baseline, while the supporting hospital-based analysis provided clinically and radiologically confirmed data from a neurosurgical population. Harmonized sleep categories and outcome definitions supported comparison across datasets, and the analytical strategy integrated sequential adjustment, nonlinear modeling, subgroup analyses with multiplicity correction, marker-specific sensitivity analyses, extended confounder adjustment, and multiple imputation of missing data. Several limitations should also be considered. Sleep duration was self-reported or abstracted from clinical records and assessed only once, which may have introduced exposure misclassification and failed to capture long-term sleep patterns, sleep quality, regularity, daytime napping, shift work, insomnia, or obstructive sleep apnea (53). In particular, long sleep may partly reflect subclinical disease that also raises inflammatory markers and sICH risk, so the long-sleep association may be affected by reverse causation or residual confounding; the 3-year landmark analysis addresses this possibility only partly, and the long-sleep estimates should be interpreted with caution. Complete-case selection excluded about 37% of eligible UK Biobank participants; although multiple imputation yielded similar estimates, selection bias cannot be fully excluded, and the healthy volunteer characteristics of UK Biobank may limit generalizability (54). Although linked health records enabled prospective outcome ascertainment, some sICH events may have been missed or misclassified (22). The hospital analysis was cross-sectional and used neurosurgical controls whose conditions may be related to both sleep and inflammation, making it susceptible to Berkson-type selection bias (19); moreover, its inflammatory measurements were obtained after sICH onset and may have been influenced by the acute hemorrhage. The composite marker score assigned equal weights to partly overlapping components, although marker-specific analyses yielded similar estimates. Although additional adjustment for antithrombotic, antihypertensive, and statin use, blood pressure, self-rated health, depression, and frailty only modestly attenuated the estimates, these factors were measured only at baseline, and residual confounding by changes in medication during follow-up, environmental exposures, sleep-disordered breathing, and other indicators of poor health remains possible (55–57). Long sleep was uncommon, resulting in wider confidence intervals, and the change-in-estimate analysis could not distinguish confounding from mediation. Future studies should incorporate repeated objective sleep measurements, systematic assessment of sleep-disordered breathing, repeated pre-event inflammatory biomarkers, adjudicated sICH phenotypes, and more diverse populations.

## 6 Conclusion

In this two-part investigation combining a large prospective UK Biobank cohort with supporting hospital-based neurosurgical data, both short and long sleep duration were associated with a higher hazard of incident sICH in the UK Biobank and with higher odds of sICH in the hospital dataset, with the strongest and most consistent associations observed for long sleep, whereas the evidence for short sleep was weaker. Dose–response analyses suggested a U-shaped relationship, with the lowest estimated hazard or odds at approximately 7 to 8 h of sleep per day. Exploratory subgroup analyses did not provide robust evidence of effect modification, including by systemic inflammatory marker burden. Additional adjustment for the inflammatory marker score modestly changed the estimates, and the data did not establish inflammation as a mediator. Collectively, these findings identify habitual sleep duration, particularly long sleep, as a potentially useful marker of sICH susceptibility and support simple sleep-habit screening in high-risk clinical populations, while further longitudinal and mechanistic studies are needed to clarify causality and determine whether improving sleep patterns can contribute to sICH prevention.

## Data Availability

The data analyzed in this study is subject to the following licenses/restrictions: Individual-level UK Biobank participant data used in this study are available to approved researchers through the UK Biobank access process and cannot be redistributed by the authors under the UK Biobank Material Transfer Agreement. Researchers may apply for access through UK Biobank (https://www.ukbiobank.ac.uk/use-our-data/apply-for-access/). The hospital-based data are not publicly available because of patient privacy regulations but are available from the corresponding authors upon reasonable request and with permission of the Ethics Committee of Jinjiang Municipal Hospital. Requests to access these datasets should be directed to PH, hopewei1888@163.com.
