# Supplementary material for: Habitual sleep duration and spontaneous intracerebral hemorrhage: a prospective UK Biobank cohort study with supporting hospital-based neurosurgical data
Source: Front Neurol. 2026 Oct 26;17:1960098. doi: 10.3389/fneur.2026.1960098 (PMC13745213; doi:10.3389/fneur.2026.1960098)
Supplement: Supplementary file 1 [file Supplementary_file_1.DOCX]

**Habitual Sleep Duration and Spontaneous Intracerebral Hemorrhage: A Prospective UK Biobank Cohort Study with Supporting Hospital-Based Neurosurgical Data**

Weichuan Dai^1^, Yuyan Zhu^1^, Liming Yang^2^, Mingming Zhang^3^, Yingxian Chen^1^, Zhongning Fang^1^, Zhen Qi^1^, Pengwei Hou^1*^, Xieli Guo^1*^

**Table of Contents**

**Text S1.** Interaction tests and false-discovery-rate correction

**Text S2.** Three-year landmark analysis excluding early sICH events

**Text S3.** Alternative inflammatory marker specifications

**Text S4.** Alternative outcome definition using broader hemorrhagic stroke

**Text S5.** Proportional hazards diagnostics for the UK Biobank Cox model

**Text S6.** Goodness of fit, calibration, and discrimination of the hospital logistic model

**Text S7.** Multicollinearity and influential observation assessments

**Text S8.** Composition of hospital controls and restricted-control sensitivity analysis

**Text S9.** Additional adjustment for intracerebral hemorrhage-specific confounders and indicators of prevalent ill health

**Text S10.** Comparison of included and excluded participants and multiple imputation of missing data

**Table S1.** Nominal and Benjamini–Hochberg-adjusted *P* values for interaction between sleep duration category and each candidate modifier.

**Table S2.** Three-year landmark sensitivity analysis in the UK Biobank.

**Table S3A.** Alternative inflammation adjustment in the UK Biobank.

**Table S3B.** Alternative inflammation adjustment in the Jinjiang Municipal Hospital analysis.

**Table S4.** Sensitivity analysis using a broader hemorrhagic stroke outcome in the UK Biobank.

**Table S5.** Covariate-specific and global proportional hazards tests for the fully adjusted UK Biobank Cox model.

**Table S6.** Model performance and calibration statistics for the most extensively adjusted Jinjiang Municipal Hospital logistic regression model.

**Table S7.** Generalized variance inflation factors for the most extensively adjusted hospital logistic model.

**Table S8.** Influence sensitivity analyses for the most extensively adjusted hospital logistic regression model.

**Table S9.** Principal diagnoses of non-ICH neurosurgical controls in the Jinjiang Municipal Hospital dataset (n = 515).

**Table S10.** Hospital-based estimates after restricting non-ICH controls to 203 elective patients with degenerative spinal disease, trigeminal neuralgia, or hemifacial spasm.

**Table S11.** Additional adjustment for intracerebral hemorrhage-specific confounders and indicators of prevalent ill health in the UK Biobank.

**Table S12.** Characteristics of included participants and of eligible participants excluded because of missing covariates or biomarkers in the UK Biobank.

**Table S13.** Multiple imputation sensitivity analysis in the UK Biobank.

**Figure S1.** Empirical receiver operating characteristic curve for the most extensively adjusted hospital logistic model, derived from the fitted probabilities of the model.

**Figure S2.** Calibration plot for the most extensively adjusted hospital logistic model.

**Figure S3.** Cook’s distance for observations in the most extensively adjusted hospital logistic model.

**Text S1.** Interaction tests and false-discovery-rate correction

Potential effect modification was assessed by testing interactions between the three-category sleep-duration variable and each candidate modifier, one at a time, in the Model III framework. For each candidate modifier, the interaction P value was obtained from a joint Wald test of all sleep-by-modifier cross-product terms, and P values were adjusted for multiple testing using the Benjamini–Hochberg procedure separately across the 15 candidate modifiers in the UK Biobank and the 13 candidate modifiers in the hospital dataset. Nominal interactions were observed for systemic inflammatory marker burden, hypertension, and age in the UK Biobank and for systemic inflammatory marker burden and age in the hospital dataset, but none remained significant after correction.

**Text S2.** Three-year landmark analysis excluding early sICH events

To reduce the possibility that preclinical disease altered habitual sleep duration before diagnosis, participants who developed sICH during the first 3 years after baseline were excluded. The landmark cohort included participants who were alive, under follow-up, and free of sICH at 3 years, and follow-up was recalculated from the landmark date. Compared with the full-cohort Model IV analysis, the estimates were modestly attenuated but remained directionally consistent, supporting the robustness of the association to exclusion of early events.

**Text S3.** Alternative inflammatory marker specifications

To evaluate whether the main findings depended on the construction of the composite systemic inflammatory marker score, Model IV was refitted after replacing the composite score with individual inflammatory markers or a binary high-marker-burden indicator. Because the neutrophil-to-lymphocyte ratio and the systemic immune-inflammation index share neutrophil and lymphocyte counts, these marker-specific models also show whether the equal weighting of partly overlapping components influenced the results. Right-skewed biomarkers were log-transformed and standardized before inclusion. Across marker-specific models, both short and long sleep remained positively associated with sICH, and long sleep consistently showed the larger estimate.

**Text S4.** Alternative outcome definition using broader hemorrhagic stroke

The UK Biobank analysis was repeated using a broader incident hemorrhagic stroke endpoint defined by ICD-10 codes I60 to I62. The broader endpoint yielded more events and slightly smaller effect estimates than the sICH-specific analysis. Nevertheless, short and long sleep remained associated with higher risk, with the larger estimate observed for long sleep.

**Text S5.** Proportional hazards diagnostics for the UK Biobank Cox model

Scaled Schoenfeld residual tests showed no statistically significant evidence that the proportional hazards assumption was violated for the sleep-duration indicators or for the model as a whole.

**Text S6.** Goodness of fit, calibration, and discrimination of the hospital logistic model

The most extensively adjusted hospital logistic model showed acceptable overall fit and moderate discrimination. The Hosmer–Lemeshow test did not indicate lack of fit. The apparent calibration intercept and slope were 0 and 1 by construction in the development sample, and bootstrap internal validation with 1,000 resamples produced an optimism-corrected calibration intercept of −0.036 and a calibration slope of 0.918. The calibration plot showed generally close agreement between observed and predicted sICH probabilities across deciles of predicted risk.

**Text S7.** Multicollinearity and influential observation assessments

All adjusted generalized variance inflation factors were below 2.2, indicating no important multicollinearity. Eight observations were flagged by at least one influence criterion, including high leverage, an absolute standardized residual greater than 3, or Cook's distance greater than 4/n. Excluding these observations produced only minor changes in the sleep-duration estimates and did not alter the substantive interpretation, and re-estimation with HC3 robust standard errors yielded identical point estimates with slightly wider confidence intervals.

**Text S8.** Composition of hospital controls and restricted-control sensitivity analysis

To assess whether the choice of neurosurgical controls influenced the hospital-based estimates, the principal hospital model (Model III) was refitted after restricting controls to elective admissions for degenerative spinal disease, trigeminal neuralgia, or hemifacial spasm. Of the 515 controls, 203 met this definition; patients with brain tumors, traumatic brain injury, hydrocephalus, unruptured cerebrovascular disorders, and other diagnoses were excluded because these conditions could independently affect sleep, systemic inflammatory markers, or acute stress. In the restricted analysis (312 sICH cases and 203 controls), the ORs were 1.47 (95% CI 0.96 to 2.26) for short sleep and 2.18 (95% CI 1.13 to 4.20) for long sleep, directionally consistent with the principal hospital estimates.

**Text S9. Additional adjustment for intracerebral hemorrhage-specific confounders and indicators of prevalent ill health**

To address potential confounding by intracerebral hemorrhage-specific risk factors and by indicators of prevalent ill health, the principal UK Biobank model (Model III) was additionally adjusted for baseline use of antiplatelet agents, oral anticoagulants, antihypertensive medication, and statins and for systolic blood pressure, calculated as the mean of the automated baseline measurements (extended model 1), and then additionally for self-rated health, depression, and frailty, the last defined using a modified Fried phenotype (extended model 2). Because blood pressure and antihypertensive treatment may lie on the pathway between sleep duration and sICH, these models were regarded as sensitivity analyses. The HRs for short and long sleep were 1.12 (95% CI 1.01 to 1.24) and 1.48 (95% CI 1.10 to 2.00) in extended model 1 and 1.10 (95% CI 0.99 to 1.23) and 1.43 (95% CI 1.04 to 1.98) in extended model 2, compared with 1.14 (95% CI 1.03 to 1.26) and 1.55 (95% CI 1.15 to 2.09) in Model III; the long-sleep association persisted, whereas the confidence interval for short sleep included unity in extended model 2 (Table S11).

**Text S10. Comparison of included and excluded participants and multiple imputation of missing data**

Of the 497,494 UK Biobank participants who were eligible after baseline exclusions, 183,258 (36.8%) were excluded from the complete-case analysis because of missing core covariates (n = 136,657) or inflammatory biomarkers (n = 46,601). Included and excluded participants were compared with respect to age, sex, sleep-duration distribution, and sICH incidence (Table S12). Missing covariate and biomarker values were then imputed by multiple imputation by chained equations with 20 imputed datasets; the imputation model included all model covariates, sleep duration, the inflammatory biomarkers, the event indicator, and the Nelson–Aalen estimate of the cumulative hazard. Models III and IV were re-estimated in all 497,494 eligible participants, and estimates were combined using Rubin’s rules (Table S13). Excluded participants were slightly older, more often reported short or long sleep, and had a higher sICH incidence than included participants (Table S12). After multiple imputation in all 497,494 eligible participants, the Model III HRs were 1.13 (95% CI 1.05 to 1.22) for short sleep and 1.50 (95% CI 1.21 to 1.87) for long sleep; the corresponding Model IV HRs were 1.09 (95% CI 1.01 to 1.18) and 1.39 (95% CI 1.11 to 1.73), respectively.

**Table S1.** Nominal and Benjamini–Hochberg-adjusted *P* values for interaction between sleep duration category and each candidate modifier.

| **Candidate modifier** | **Nominal *P* for interaction** | **Benjamini–Hochberg-adjusted *P* value** |
| --- | --- | --- |
| **UK Biobank (15 candidate modifiers)** |  |  |
| Systemic inflammatory marker burden | 0.008 | 0.113 |
| Hypertension | 0.015 | 0.113 |
| Age group | 0.037 | 0.185 |
| Diabetes | 0.064 | 0.240 |
| Smoking status | 0.091 | 0.273 |
| Physical activity | 0.118 | 0.295 |
| Body mass index | 0.146 | 0.313 |
| Charlson comorbidity index | 0.181 | 0.339 |
| Sex | 0.227 | 0.378 |
| Educational attainment | 0.264 | 0.396 |
| Income level | 0.317 | 0.432 |
| Alcohol drinking status | 0.356 | 0.445 |
| Residence | 0.421 | 0.486 |
| Townsend deprivation index | 0.488 | 0.523 |
| Race/ethnicity | 0.612 | 0.612 |
| **Jinjiang Municipal Hospital (13 candidate modifiers)** |  |  |
| Systemic inflammatory marker burden (admission) | 0.041 | 0.267 |
| Age group | 0.039 | 0.267 |
| Hypertension | 0.077 | 0.334 |
| Smoking status | 0.109 | 0.343 |
| Physical activity | 0.132 | 0.343 |
| Diabetes | 0.168 | 0.364 |
| Body mass index | 0.201 | 0.373 |
| Charlson comorbidity index | 0.248 | 0.403 |
| Sex | 0.291 | 0.420 |
| Educational attainment | 0.344 | 0.447 |
| Income level | 0.389 | 0.460 |
| Alcohol drinking status | 0.463 | 0.502 |
| Residence | 0.584 | 0.584 |

Interaction *P* values were obtained from joint Wald tests in the Model III framework; in the hospital dataset, inflammatory markers were measured at admission, after sICH onset in cases.

**Table S2.** Three-year landmark sensitivity analysis in the UK Biobank.

| **Analysis** | **Participants / events** | **Short sleep, HR (95% CI)** | **Long sleep, HR (95% CI)** |
| --- | --- | --- | --- |
| Full-cohort Model IV analysis | 314,236 / 1,387 | 1.10 (1.01 to 1.20) | 1.42 (1.06 to 1.90) |
| Three-year landmark analysis | 306,941 / 1,196 | 1.09 (1.00 to 1.19) | 1.39 (1.02 to 1.89) |

Normal sleep duration of 7 to 9 h/day was the reference. Models used the Model IV covariate set, including the continuous systemic inflammatory marker score.

**Table S3A.** Alternative inflammation adjustment in the UK Biobank.

| **Inflammation adjustment** | **Short sleep, HR (95% CI)** | **Long sleep, HR (95% CI)** |
| --- | --- | --- |
| Composite inflammatory marker score | 1.10 (1.01 to 1.20) | 1.42 (1.06 to 1.90) |
| C-reactive protein | 1.12 (1.02 to 1.23) | 1.48 (1.10 to 1.99) |
| Neutrophil-to-lymphocyte ratio | 1.11 (1.01 to 1.21) | 1.45 (1.08 to 1.95) |
| Platelet-to-lymphocyte ratio | 1.13 (1.03 to 1.24) | 1.51 (1.12 to 2.03) |
| Systemic immune-inflammation index | 1.11 (1.02 to 1.22) | 1.46 (1.09 to 1.97) |
| High marker burden, top quartile | 1.12 (1.02 to 1.23) | 1.49 (1.11 to 2.00) |

Normal sleep duration was the reference.

**Table S3B.** Alternative inflammation adjustment in the Jinjiang Municipal Hospital analysis.

| **Inflammation adjustment** | **Short sleep, OR (95% CI)** | **Long sleep, OR (95% CI)** |
| --- | --- | --- |
| Composite inflammatory marker score | 1.34 (1.01 to 1.79) | 1.76 (1.02 to 3.04) |
| C-reactive protein | 1.41 (1.04 to 1.91) | 1.91 (1.08 to 3.38) |
| Neutrophil-to-lymphocyte ratio | 1.38 (1.02 to 1.87) | 1.84 (1.04 to 3.26) |
| Platelet-to-lymphocyte ratio | 1.44 (1.06 to 1.96) | 1.98 (1.11 to 3.52) |
| Systemic immune-inflammation index | 1.39 (1.03 to 1.89) | 1.87 (1.05 to 3.32) |
| High marker burden, top quartile | 1.40 (1.03 to 1.90) | 1.89 (1.07 to 3.35) |

Normal sleep duration was the reference. Inflammatory markers were measured at admission, after sICH onset in cases; these analyses are exploratory.

**Table S4.** Sensitivity analysis using a broader hemorrhagic stroke outcome in the UK Biobank.

| **Outcome definition** | **Incident events** | **Short sleep, HR (95% CI)** | **Long sleep, HR (95% CI)** |
| --- | --- | --- | --- |
| Spontaneous intracerebral hemorrhage | 1,387 | 1.10 (1.01 to 1.20) | 1.42 (1.06 to 1.90) |
| Broader hemorrhagic stroke, I60 to I62 | 2,416 | 1.08 (1.02 to 1.15) | 1.34 (1.10 to 1.63) |

Models were adjusted for the Model IV covariate set.

**Table S5.** Covariate-specific and global proportional hazards tests for the fully adjusted UK Biobank Cox model.

| **Model IV term** | **Schoenfeld residual test *P* value** |
| --- | --- |
| Short sleep | 0.438 |
| Long sleep | 0.367 |
| Age | 0.158 |
| Sex | 0.552 |
| Race/ethnicity | 0.614 |
| Body mass index | 0.231 |
| Educational attainment | 0.486 |
| Income level | 0.657 |
| Townsend deprivation index | 0.409 |
| Residence | 0.738 |
| Smoking status | 0.297 |
| Alcohol drinking status | 0.719 |
| Physical activity | 0.604 |
| Charlson comorbidity index | 0.113 |
| Diabetes | 0.476 |
| Hypertension | 0.194 |
| Systemic inflammatory marker score | 0.247 |
| Global test | 0.281 |

**Table S6.** Model performance and calibration statistics for the most extensively adjusted Jinjiang Municipal Hospital logistic regression model.

| **Diagnostic measure** | **Estimate** | **Additional information** |
| --- | --- | --- |
| Hosmer–Lemeshow test | χ^2^ = 7.18, df = 8 | *P* = 0.517 |
| Apparent calibration intercept | 0.000 | Development-sample value |
| Apparent calibration slope | 1.000 | Development-sample value |
| Bootstrap-corrected calibration intercept | −0.036 | 1,000 bootstrap resamples |
| Bootstrap-corrected calibration slope | 0.918 | 1,000 bootstrap resamples |
| Brier score | 0.1763 | Lower values indicate better overall accuracy |
| Area under the ROC curve | 0.7806 | DeLong 95% CI 0.7451 to 0.8158 |

Abbreviations: CI, confidence interval; df, degrees of freedom; ROC, receiver operating characteristic.

**Table S7.** Generalized variance inflation factors for the most extensively adjusted hospital logistic model.

| **Model IV term** | **Df** | **GVIF^(1/(2×Df))** |
| --- | --- | --- |
| Age | 1 | 1.44 |
| Sex | 1 | 1.17 |
| Body mass index | 1 | 1.35 |
| Educational attainment | 2 | 1.26 |
| Income level | 2 | 1.29 |
| Residence | 1 | 1.21 |
| Smoking status | 2 | 1.28 |
| Alcohol drinking status | 2 | 1.22 |
| Physical activity | 1 | 1.14 |
| Charlson comorbidity index | 1 | 2.16 |
| Diabetes | 1 | 1.47 |
| Hypertension | 1 | 1.81 |
| Systemic inflammatory marker score | 1 | 2.09 |
| Short sleep | 1 | 1.08 |
| Long sleep | 1 | 1.06 |

Abbreviations: Df, degrees of freedom; GVIF, generalized variance inflation factor.

**Table S8.** Influence sensitivity analyses for the most extensively adjusted hospital logistic regression model.

| **Analysis** | **Participants / sICH cases** | **Short sleep, OR (95% CI)** | **Long sleep, OR (95% CI)** |
| --- | --- | --- | --- |
| Hospital Model IV | 827 / 312 | 1.34 (1.01 to 1.79) | 1.76 (1.02 to 3.04) |
| After excluding 8 potentially influential observations | 819 / 309 | 1.31 (0.97 to 1.78) | 1.73 (1.01 to 2.98) |
| Model IV with HC3 robust standard errors | 827 / 312 | 1.34 (0.99 to 1.82) | 1.76 (1.00 to 3.11) |

**Table S9.** Principal diagnoses of non-ICH neurosurgical controls in the Jinjiang Municipal Hospital dataset (n = 515).

| **Principal diagnosis** | **n (%)** |
| --- | --- |
| Brain tumor without hemorrhage | 141 (27.4) |
| Traumatic brain injury without intracranial hemorrhage | 63 (12.2) |
| Degenerative spinal disease | 119 (23.1) |
| Functional neurosurgical disorders (trigeminal neuralgia or hemifacial spasm) | 84 (16.3) |
| Hydrocephalus | 31 (6.0) |
| Unruptured cerebrovascular disorders | 36 (7.0) |
| Other conditions | 41 (8.0) |
| Total | 515 (100.0) |

**Table S10.** Hospital-based estimates after restricting non-ICH controls to 203 elective patients with degenerative spinal disease, trigeminal neuralgia, or hemifacial spasm.

| **Analysis** | **Participants / sICH cases** | **Short sleep, OR (95% CI)** | **Long sleep, OR (95% CI)** |
| --- | --- | --- | --- |
| Principal hospital model (Model III), all controls | 827 / 312 | 1.49 (1.04 to 2.13) | 2.05 (1.17 to 3.59) |
| Restricted elective controls | 515 / 312 | 1.47 (0.96 to 2.26) | 2.18 (1.13 to 4.20) |

Normal sleep duration was the reference; models were adjusted for the Model III covariates.

**Table S11.** Additional adjustment for intracerebral hemorrhage-specific confounders and indicators of prevalent ill health in the UK Biobank.

| **Model** | **Participants / events** | **Short sleep, HR (95% CI)** | **Long sleep, HR (95% CI)** |
| --- | --- | --- | --- |
| Model III (principal model) | 314,236 / 1,387 | 1.14 (1.03 to 1.26) | 1.55 (1.15 to 2.09) |
| Extended model 1: Model III + antiplatelet agents, oral anticoagulants, antihypertensive medication, statins, and systolic blood pressure | 307,146 / 1,356 | 1.12 (1.01 to 1.24) | 1.48 (1.10 to 2.00) |
| Extended model 2: extended model 1 + self-rated health, depression, and frailty | 281,973 / 1,244 | 1.10 (0.99 to 1.23) | 1.43 (1.04 to 1.98) |

Normal sleep duration (7–9 h/day) was the reference. Extended models were fitted in participants with complete data on the additional covariates.

**Table S12.** Characteristics of included participants and of eligible participants excluded because of missing covariates or biomarkers in the UK Biobank.

| **Characteristic** | **Included participants (n = 314,236)** | **Excluded participants (n = 183,258)** |
| --- | --- | --- |
| Age, years, mean (SD) | 56.6 (8.0) | *57.8 (8.7)* |
| Female, n (%) | 171,236 (54.5) | 99,509 (54.3) |
| Short sleep, <7 h/day, n (%) | 77,783 (24.8) | 49,176 (26.8) |
| Normal sleep, 7–9 h/day, n (%) | 230,812 (73.5) | 128,591 (70.2) |
| Long sleep, >9 h/day, n (%) | 5,641 (1.8) | 5,491 (3.0) |
| Incident sICH events, n | 1,387 | 1,149 |
| sICH incidence per 1,000 person-years | 0.32 | 0.46 |

Excluded participants were eligible after baseline exclusions but lacked data on core covariates (n = 136,657) or inflammatory biomarkers (n = 46,601).

**Table S13.** Multiple imputation sensitivity analysis in the UK Biobank.

| **Analysis** | **Participants / events** | **Short sleep, HR (95% CI)** | **Long sleep, HR (95% CI)** |
| --- | --- | --- | --- |
| Complete-case analysis, Model III | 314,236 / 1,387 | 1.14 (1.03 to 1.26) | 1.55 (1.15 to 2.09) |
| Complete-case analysis, Model IV | 314,236 / 1,387 | 1.10 (1.01 to 1.20) | 1.42 (1.06 to 1.90) |
| Multiple imputation, Model III | 497,494 / 2,536 | 1.13 (1.05 to 1.22) | 1.50 (1.21 to 1.87) |
| Multiple imputation, Model IV | 497,494 / 2,536 | 1.09 (1.01 to 1.18) | 1.39 (1.11 to 1.73) |

Normal sleep duration (7–9 h/day) was the reference. Multiple imputation by chained equations (20 imputed datasets) included all model covariates, sleep duration, the inflammatory biomarkers, the event indicator, and the Nelson–Aalen estimate of the cumulative hazard; estimates were combined using Rubin’s rules. In each imputed dataset, the inflammatory marker score was recalculated from the imputed biomarker components using the same transformations and standardization procedure as in the complete-case analysis.

**Figure S1.** Empirical receiver operating characteristic curve for the most extensively adjusted hospital logistic model, derived from the fitted probabilities of the model.

**Figure S2.** Calibration plot for the most extensively adjusted hospital logistic model. Points represent observed sICH proportions within deciles of predicted probability, and the dashed line denotes perfect calibration. Optimism-corrected calibration statistics from 1,000 bootstrap resamples are reported in Table S6.

**Figure S3.** Cook’s distance for observations in the most extensively adjusted hospital logistic model. The dashed line denotes the prespecified screening threshold of 4/n (0.00484). Orange points indicate the eight observations flagged by at least one prespecified influence criterion (Cook’s distance, leverage, or standardized residual).
